# Cholangitis with bacteremia due to *Pseudomonas nitroreducens* in a patient with pancreatic neuroendocrine tumors: a case report

**DOI:** 10.1186/s12879-024-09092-8

**Published:** 2024-02-09

**Authors:** Naoya Itoh, Nana Akazawa, Makoto Yamaguchi, Hiromi Murakami, Kiyofumi Ohkusu

**Affiliations:** 1 Division of Infectious Diseases, Aichi Cancer Center, 1-1 Kanokoden, Chikusa-ku, Nagoya, Aichi 464-8681 Japan; 2 Department of Microbiology, Tokyo Medical University, 6-1-1 Shinjuku-ku, Shinjuku, Tokyo, 160-8402 Japan

**Keywords:** *Pseudomonas nitroreducens*, Cholangitis, MALDI-TOF MS, 16S rRNA sequencing, Case report

## Abstract

**Background:**

*Pseudomonas nitroreducens* is a non-fermenting, gram-negative, rod-shaped bacterium commonly inhabiting soil, particularly soil contaminated with oil brine. To our knowledge, no cases of human infection with *P. nitroreducens* have been previously reported. Here, we present the first documented case of cholangitis caused by *P. nitroreducens* in a patient with bacteremia.

**Case presentation:**

A 46-year-old Japanese man with an advanced pancreatic neuroendocrine tumor was hospitalized with fever and chills. Four days before admission, the patient developed right upper abdominal pain. Two days later, he also experienced fever and chills. Endoscopic retrograde cholangiopancreatography was performed on the day of admission, and the patient was diagnosed as having cholangitis associated with stent dysfunction. Gram-negative rods were isolated from blood cultures, but attempts to identify the bacteria using VITEK2 and matrix-assisted laser desorption/ionization time-of-flight mass spectrometry (MALDI-TOF MS) with VITEK MS ver. 4.7.1 (bioMérieux Japan Co. Ltd., Tokyo, Japan) were unsuccessful. Finally, the organism was identified as *P. nitroreducens* using MALDI-TOF MS with a MALDI Biotyper (Bruker Daltonics Co., Ltd., Billerica, MA, USA) and 16 S ribosomal RNA sequencing. Despite thorough interviews with the patient, he denied any exposure to contaminated soil. The patient was treated with intravenous cefepime and oral ciprofloxacin for 16 days based on susceptibility results, achieving a good therapeutic outcome. At the outpatient follow-up on day 28, the patient was in good general condition.

**Conclusions:**

This is the first reported human case of cholangitis with bloodstream infection caused by *P. nitroreducens*. This report provides clinicians with novel insights into the clinical manifestations and diagnostic methods necessary for the accurate diagnosis of *P. nitroreducens*, along with guidance on treatment.

## Background

*Pseudomonas nitroreducens* is a gram-negative, motile, aerobic, rod-shaped, synchrotrophic bacterium inhabiting soils such as oil brines, paddy field drainage, and river sediments [1–3]. *P. nitroreducens* is well-known for its ability to synthesize polyhydroxybutyrate homopolymers (polyesters) from medium-chain fatty acids, a feature used in the industrial production of polyesters [4]. *P. nitroreducens* has been identified in the bronchoalveolar lavage fluid of patients with multiple myeloma [5], respiratory specimens from patients with cystic fibrosis [6], and ileal biopsies from pediatric patients [7]. However, these previous studies reported on cases of colonization, and to the best of our knowledge, there have been no reports of human infection. In addition, no cases of infection in animals have been reported. Here, we report the first human infection with *P. nitroreducens* in a patient with cholangitis and bloodstream infection.

## Case presentation

A 46-year-old Japanese man with an advanced pancreatic neuroendocrine tumor presented with right upper abdominal pain and later also experienced fever and chills. A pancreatic neuroendocrine tumor was treated with pancreaticoduodenectomy approximately 10 years earlier, and multiple liver metastases were identified intraoperatively. The patient was treated postoperatively with a chemotherapy regimen of everolimus for 1 year. Subsequently, lanreotide was administered for 3 months as part of a clinical trial. However, his liver metastases worsened upon switching to lanreotide, necessitating a return to everolimus treatment, with which he has been treated for approximately 9 years. He had a history of multiple episodes of cholangitis and stenosis of the choledochojejunostomy, for which a plastic stent was placed.

Four days before admission, the patient developed right upper abdominal pain, followed by a fever of 38.4 °C and chills 2 days later. Upon examination, he appeared in good condition, with a temperature of 36.7 °C, a regular heart rate of 104 bpm, a blood pressure of 139/82 mmHg, a respiratory rate of 16 bpm, and an oxygen saturation of 97% on room air. His abdomen was flat and soft, with mild tenderness in the right upper quadrant. The other physical examination results were unremarkable. Laboratory investigation results were as follows: white blood cell count 8,040/mL (neutrophil count: 5,740/mL), hemoglobin 14.2 g/dL, C-reactive protein 11.28 mg/dL (normal < 0.30 mg/dL), gamma-glutamyltransferase 136 U/L (normal < 30 U/L), alkaline phosphatase 283 U/L (normal 115–359 U/L), total bilirubin 0.5 mg/dL (normal 0.3–1.2 mg/dL), and direct bilirubin 0.2 mg/dL (normal 0.0–0.2 mg/dL).

Two sets of blood culture samples were obtained from peripheral blood and collected in BacT/ALERT FA PLUS culture bottles using the BacT/ALERT 3D system (BioMérieux Japan Co. Ltd., Tokyo, Japan). Empirical antimicrobial therapy with intravenous cefmetazole (CMZ) (3 g/day) was instituted for cholangitis. Endoscopic retrograde cholangiopancreatography performed on the day of admission (day 0) revealed stenosis in the anterior segment branch, followed by anastomotic dilation. The stent was removed because of recurring episodes of stent dysfunction, suspected to be caused by the stent placement itself. No bile cultures were collected during this procedure.

**Fig. 1:**
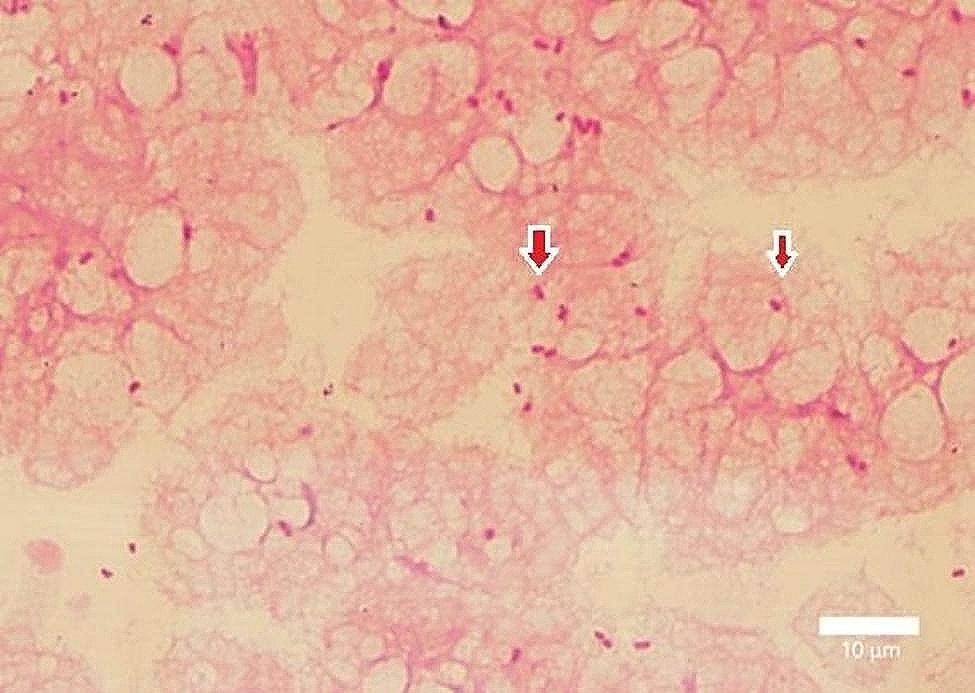
A smear of blood culture demonstrating gram-negative bacilli (red arrow). Gram staining, 1,000 × (300 dpi). (This image was acquired and captured using a Nikon Eclipse 55i microscope (Nikon, Tokyo, Japan) and a Nikon Digital Color Camera Sight DS-Fi-1 (Nikon)

**Fig. 2:**
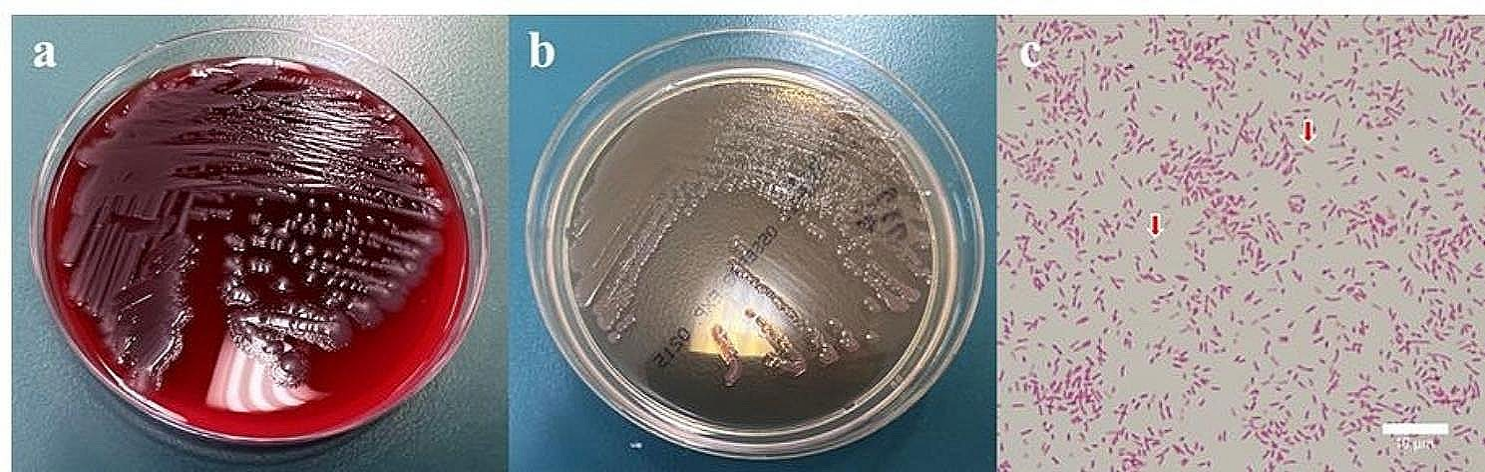
White colonies of *Pseudomonas nitroreducens* after 24 h on sheep blood agar plate (**a**). Lactose-negative colonies of *P. nitroreducens* on a MacConkey II agar plate (**b**). A smear of the colonies showing gram-negative rods (red arrow). Gram staining, 1000 × (300 dpi). (These images were acquired and captured using a Nikon Eclipse 55i microscope (Nikon) and a Nikon Digital Color Camera Sight DS-Fi-1 (Nikon)

Gram-negative rods were identified on examination of the culture of the blood samples from both sets of aerobic bottles collected upon admission at 18 h and 59 min and 19 h and 29 min, respectively (Fig. 1). CMZ was changed to intravenous cefepime (CFPM) at a dose of 3 g/day owing to the possibility of non-fermenting glucose bacteria (day 3). Positive bottles were subcultured on sheep blood agar plates (Nissui Pharmaceuticals Co., Ltd., Tokyo, Japan) and MacConkey II agar plates (Becton Dickinson Co., Ltd., Franklin Lakes, NJ, USA). After 24 h of aerobic incubation at 35 °C, non-hemolytic white colonies of gram-negative bacilli were observed on the sheep blood agar plates and lactose-negative colonies of gram-negative bacilli on MacConkey II agar plates (Fig. 2a-c).

Attempts to identify bacteria using VITEK2 and matrix-assisted laser desorption/ionization time-of-flight mass spectrometry (MALDI-TOF MS) with VITEK MS ver. 4.7.1 (bioMérieux Japan Co. Ltd., Tokyo, Japan) were unsuccessful. Identification was achieved using MALDI-TOF MS with a MALDI Biotyper (Bruker Daltonics Co., Ltd., Billerica, MA, USA) and 16 S ribosomal RNA (rRNA) sequencing. The isolated strain was evaluated using MALDI Biotyper version 12, demonstrating *P. nitroreducens* with a 2.11 score value. The isolated colony from the blood culture was used to perform a polymerase chain reaction (PCR) using universal primers 27 F and 1492R. The primer sequences were 27 F 5′-AGAGTTTGATCMTGGCTCAG-3′ and 1492R 5′-CGGTTACCTTGTTACGACTT-3′. The PCR products were run on a gel, and the eluted DNA was further purified and sequenced. After performing a Basic Local Alignment Search Tool (BLAST) for the 16 S rRNA sequence of the isolated strain, 99.66% homology (1451/1456 bp) with the standard *P. nitroreducens* strain DSM 14,399 (T) (GenBank Accession No.: AM088474) was identified. Owing to the 99.52% homology (1450/1457 bp) with the standard strain, *P. nicosulfuronedens* LAM1902 (T) (GenBank Accession No.: MN007089), a urease test was performed to distinguish *P. nitroreducens* from the standard strain [8]. Owing to the positive result of the urease test, the organism identified in the blood culture was confirmed to be *P. nitroreducens*.

The patient was diagnosed as having cholangitis and bacteremia caused by *P. nitroreducens*. Antimicrobial susceptibility tests were performed according to the methodology recommended by the Clinical and Laboratory Standards Institute document M100-Ed 33 (2023) for other non-Enterobacteriaceae isolates as there are no interpretive criteria for this strain. The *P. nitroreducens* isolates from blood cultures were susceptible to CFPM (Table 1).

**Table 1:** Antibiotic susceptibility of isolated *Pseudomonas nitroreducens*

	MIC (µg/mL)	Susceptibility
Piperacillin	16	S
Piperacillin tazobactam	16	S
Ceftazidime	4	S
Cefepime	≤ 1	S
Ceftriaxone	16	I
Imipenem	≤ 0.25	S
Meropenem	≤ 0.25	S
Gentamicin	≤ 1	S
Amikacin	4	S
Minocycline	≥ 16	R
Ciprofloxacin	≤ 0.25	S
Levofloxacin	1	S
Trimethoprim-sulfamethoxazole	80	R

Abbreviations: MIC, minimal inhibitory concentration; S, susceptible; I, intermediate; R, resistant

**Fig. 3:**
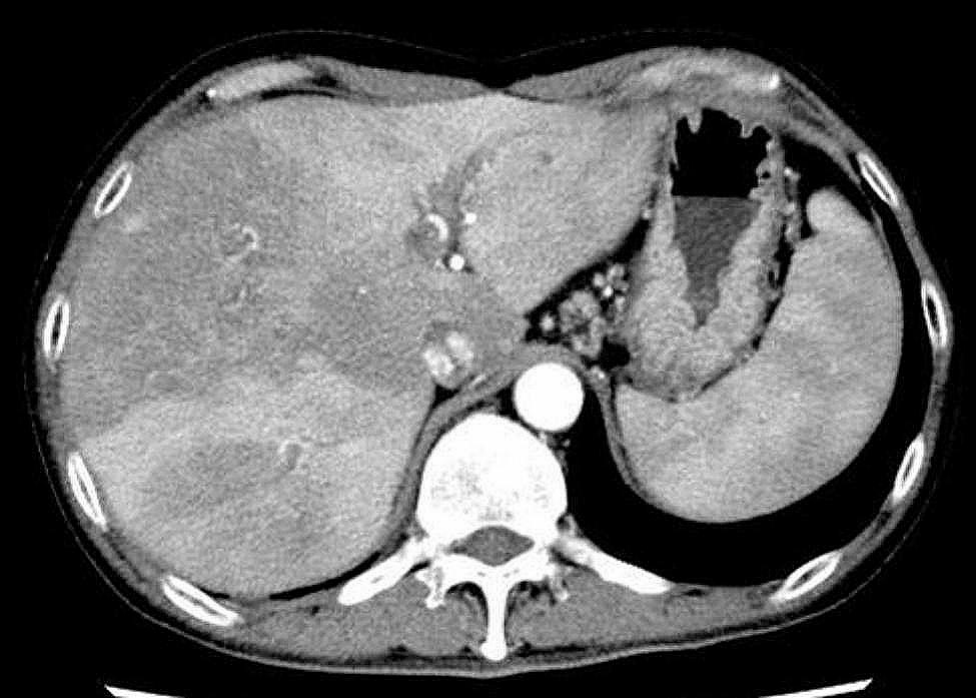
Computed tomography showing early inhomogeneous enhancement of the liver

A thorough and careful interview of the patient revealed that he was deskbound and had no exposure to paddy drainage, oil field brine, or soil. On day 4, owing to persistent fever, contrast-enhanced computed tomography (CT) of the abdomen revealed early nonhomogeneous liver enhancement, consistent with cholangitis (Fig. 3). Subsequently, his fever subsided. On day 6, he was switched to oral ciprofloxacin 400 mg/day and was discharged from our hospital. The duration of antimicrobial therapy was 16 days. At the outpatient follow-up on day 28, the patient was in good general condition, had no fever, and was confirmed to be cured of cholangitis. Written informed consent was obtained from the patient for the publication of this case report and the accompanying images.

## Discussion and conclusions

Here, we report a case of cholangitis with bloodstream infection caused by *P. nitroreducens* in a patient with advanced pancreatic neuroendocrine tumors. To our knowledge, this is the first report of *a P. nitroreducens* infection in humans. *P. nitroreducens* could not initially be identified using VITEK2 and VITEK MS and was finally confirmed via MALDI Biotyper and 16 S rRNA sequencing.

The patient presented with cholangitis associated with a bile duct stent dysfunction. It is unknown whether *P. nitroreducens* was present in the bile because bile cultures were not collected in this case. However, since there was no other obvious focus of infection in this case and contrast-enhanced CT showed early enhancement of the liver [9], a diagnosis of cholangitis due to *P. nitroreducens* was established.

*P. nitroreducens* is a soil-dwelling organism found in oil brine, paddy field drainage, and river sediments. However, this patient was an office worker without exposure to these sources [1–3]. This suggests that *P. nitroreducens* occurs in environments other than those previously reported.

MALDI-TOF MS is a rapid, accurate, cost-effective method for identifying various organisms. However, a case was previously reported in which the pathogen was misidentified as *P. aeruginosa* via MALDI-TOF MS and was ultimately diagnosed as *P. nitroreducens* using 16 S rRNA sequencing [5]. In the present case, *P. nitroreducens* was identified via MALDI-TOF MS using the MALDI Biotyper with a score value of 2.11 and was confirmed through 16 S rRNA sequencing. The widespread use of MALDI-TOF MS has revolutionized clinical microbiological diagnosis. However, it is considered unreliable for identifying bacteria not found in libraries. This is because identification using MALDI-TOF MS requires the registration of a representative strain in the reference spectrum of the database. We also experienced difficulties in identifying *P. nitroreducens* using VITEK MS. Accurate identification of the organism is important for characterizing the clinical manifestations caused by rare microbes.

The recommended optimal treatment duration for acute cholangitis is 4–7 days once source control is achieved [10]. Concerns remained regarding drainage in this case. Thus, the patient was treated with intravenous CFPM for 3 days and then switched to oral ciprofloxacin for 13 days, for a total of 16 days, a longer treatment period. In a previous case report of *P. nitroreducens*, antimicrobial susceptibility results showed full susceptibility to amikacin, ciprofloxacin, gentamicin, tobramycin, ceftazidime, and piperacillin tazobactam but intermediate susceptibility to CFPM and meropenem [5]. Therefore, ciprofloxacin is an appropriate empirical or definitive treatment for *P. nitroreducens* infections.

In conclusion, we report the first human case of cholangitis with bacteremia due to *P. nitroreducens* with good therapeutic outcomes. This case demonstrates the need for more accurate identification of pathogens through advanced diagnostics using molecular biological techniques in infections caused by rare microorganisms to guide treatment and provide appropriate follow-up.

## Data Availability

The data used and analyzed in the current study are available from the corresponding author upon reasonable request.

## Abbreviations

CMZ: Cefmetazole
CFPM: Cefepime
MALDI-TOF MS: Matrix-assisted laser desorption/ionization time-of-flight mass spectrometry
PCR: Polymerase chain reaction
BLAST: Basic Local Alignment Search Tool
CT: Computed tomography
rRNA: ribosomal RNA
MIC: Minimal inhibitory concentration
